# Configuration, Not Content: A Network Analysis of Gendered Prototypes of Intimate Partner Violence

**DOI:** 10.1177/08862605261444009

**Published:** 2026-06-26

**Authors:** Hanna Feige, Timothy J. Luke, Karl Ask

**Affiliations:** 1 University of Gothenburg, Sweden

**Keywords:** intimate partner violence, IPV, same-sex relationships, network analysis, gendered prototypes

## Abstract

Research on perceptions of intimate partner violence (IPV) has predominantly focused on heterosexual relationships with male perpetrators and female victims. The scant research focusing on same-sex couples, and relationships with female abusers and male victims, has produced inconsistent findings that are hard to integrate. To bring more coherence to this literature, an improved theoretical understanding of people’s beliefs and assumptions about IPV is necessary. Thus, we set out to examine the structure of perceived IPV prototypes of various victim–perpetrator gender combinations. Using a mixed-methods approach, a U.S.- and U.K.-based sample (*N* = 161) generated open-ended descriptions of the victim, the perpetrator, their relationship and the abuse in four IPV scenarios (male-on-female, male-on-male, female-on-male, female-on-female). The responses were thematically coded and modeled as network structures, allowing us to study which features (e.g., stereotypes, assumptions) were present in each prototype and how these features were connected. The findings show surprisingly strong similarities in the overall structure of the prototypes across scenarios, yet strikingly clear differences in how specific features are linked in the different gender combinations. The results suggest that people’s gendered assumptions about IPV are reflected less in entirely different IPV ‘stories’ and rather in differences in how shared features are combined.

## Introduction

Research on perceptions of intimate partner violence (IPV) has predominantly centered on heterosexual IPV, specifically cases involving male perpetrators and female victims. These cases are considered ‘typical’ in the public perception, despite the prevalence of IPV being similar for both heterosexual and same-sex couples (Dagenbrink et al., 2023; Whitehead et al., 2021). The differential perception of ‘typical’ and ‘atypical’ cases is not limited to differences in expected prevalence. Research persistently finds that same-sex IPV is perceived as less severe than ‘typical’ cases, and incidents involving a male victim and a female perpetrator are considered the least severe (Savage et al., 2022; Stanziani et al., 2018). Previous research has examined factors such as beliefs about masculinity and physical differences between victim and perpetrator as potential explanations for these differences in perceived severity (Parker et al., 2022; Russell et al., 2019; Yamawaki et al., 2018). However, this research has produced inconsistent findings.

One explanation for this incoherence might be the lack of theoretical understanding of people’s beliefs and expectations about ‘atypical’ IPV. That is, how they imagine IPV when it does not involve a male perpetrator and a female victim. Without such a framework, aspects that are unique to these gender combinations are easily overlooked, which in turn hampers the development of more targeted research and interventions. Although research on these perceived ‘atypical’ cases has been growing in recent years, a cohesive theoretical and methodological approach to understanding such IPV has not yet taken shape (Calton et al., 2016). This lack of theoretical attention to other forms of IPV is mirrored in the dominant approach to studying IPV perceptions, which begins from the assumptions attached to male-on-female IPV and applies them to other gender combinations (Ahmed et al., 2013; Harris & Cook, 1994; Wise & Bowman, 1997). As a result, the ways in which studies have described IPV scenarios and constrained participants’ responses have tended to reproduce that ‘typical’ prototype rather than uncover features that might be unique to other constellations. In contrast, we propose a data-driven approach, where studies are based on the structure and content of people’s actual IPV beliefs, as identified in exploratory research. To this end, the current study used a psychometric network-modeling approach to represent prototypical beliefs about the characteristics of various typical and atypical forms of IPV.

### Gendered Perceptions of IPV

There have been many proposed explanations for the consistent finding that the genders of the perpetrator and the victim influence how severe an IPV incident is perceived to be, even while holding constant the behavior entailed in the abuse (Ahmed et al., 2013; Russell, 2018; Savage et al., 2022). The perceived severity of the incident is highest for scenarios in which the perpetrator is male and for those in which the victim is female. This pattern creates a hierarchy of severity ratings, such that male-on-female scenarios are perceived to be the most severe, followed by the same-sex scenarios, with the female-on-male scenario at the bottom (Ahmed et al., 2013; Cormier & Woodworth, 2008; Hamby & Jackson, 2010; Harris & Cook, 1994; Poorman et al., 2003; Russell, 2018; Savage et al., 2022; S. M. Seelau & Seelau, 2005; E. P. Seelau et al., 2003; Stanziani et al., 2018).

Other findings in the literature are less consistent. Some studies find a gender-based difference in the assignment of responsibility (Russell et al., 2019), while other studies fail to find such differences (Goodson, 2023; S. M. Seelau & Seelau, 2005). Some researchers have argued that the physical differences between men and women can explain the gender-based perceptions (Hamby & Jackson, 2010). Such differences could explain why a man’s perpetrated abuse in an ambiguous scenario is considered worse, even when the abuse is primarily—and more severely—perpetrated by the woman (Hine et al., 2022). However, they cannot explain why the gendered perceptions of severity seem to be unrelated to the type of abuse (physical/psychological; Ahmed et al., 2013). Research examining victim blaming (MacNeil et al., 2024; Russell, 2018) or the perceived need for outside intervention (Brown & Groscup, 2009; Stanziani et al., 2018) as explanations of gender-based differences have produced similarly inconsistent findings.

To better understand the lack of consistency in the research, it is worth looking at the methods used in previous studies. Notably, in all but two of the studies mentioned above—Savage et al. (2022) and MacNeil et al. (2024)—vignettes of IPV scenarios are presented to groups of participants, and only the genders of the victim and/or the perpetrator are varied across the scenarios. Most of the vignettes used are identical or similar to vignettes used in previous studies, and many draw directly from the materials of the first experimental study to examine these gender-based differences (Harris & Cook, 1994). Even in their landmark study, Harris and Cook adapted a version of the ‘wife-battering-scenario’ from earlier work by Kristiansen and Giulietti (1990). Thus, the ‘typical’ case of male-on-female violence has served as the reference point for the stimulus materials used in most empirical work on the perceptions of ‘atypical’ IPV. In doing so, the primary focus often lies on identifying differences between typical and atypical IPV, rather than exploring the unique characteristics of the atypical cases. For example, explanations that focus on physical differences assume that what distinguishes ‘typical’ from ‘atypical’ IPV is the perception that men can do more harm to women than to other men, and that women, in turn, can do less harm to their partners. This approach may neglect alternative explanations that are specific to ‘atypical’ IPV. For instance, stereotypical narratives such as the ‘lesbian utopia’ myth (i.e., the notion that women-only environments are inherently safe; see Barnes, 2011) may inform perceptions of female-on-female IPV as being less severe. Thus, an approach allowing for more spontaneous participant responses may provide greater insight into people’s perceptions of IPV. A better theoretical understanding of these perceptions can facilitate the development of more targeted interventions, such as training for service providers who work with victims and perpetrators of IPV.

### Hypotheses and Aims

The present study aimed to address the above limitation of past research, using a data-driven approach to accessing mental representations of IPV prototypes. By ‘data-driven’, we mean that no pre-written IPV vignettes or other descriptive materials are provided to participants. By avoiding IPV scenario descriptions that may already be imbued with gendered meanings, the study allows participants’ spontaneous understandings to shape the data. Participants were given open-ended prompts to describe different aspects of IPV scenarios. The responses were then coded to create psychometric network models of the respective prototypes. Several parameters can inform us about a network structure. Global strength refers to the overall connectivity of a network, representing the total magnitude of associations. Similarly, local strength measures the connectivity of individual nodes, indicating how strongly a variable is linked to others. Density measures the extent to which the nodes are interconnected.

As the male-on-female IPV scenario is perceived to be the ‘typical’ scenario, it seems to be the most familiar in both the public perception and the scientific discourse. We aimed to test this assumption by comparing the network strength and density of the male-on-female prototype to the other scenarios. Higher strength and density suggest more cohesiveness of the network, and in this case, of the prototype. Thus, we expected that the male-on-female scenario is the most familiar (‘typical’) scenario, featuring greater global strength and higher density than the other prototypes (H^1.1^, H^1.2^). Ratings of difficulty in rendering responses were also compared across scenarios as an additional measure of their familiarity. We expected that the male-on-female scenario would be rated as the least difficult to answer, compared to the other scenarios (H^1.3^).

The male-on-female scenario being rated the most severe is a consistent finding in the literature (Cormier & Woodworth, 2008; Savage et al., 2022). This led to the assumption that physical abuse would be more central (i.e., feature greater strength) in the network model of the male-on-female prototype, compared to the other prototypes (H^2^). Besides actual gender, the gendered traits of the persons involved also seem to influence judgments of responsibility (Russell et al., 2019). Female perpetrators who are feminine-presenting are typically perceived to be less responsible than female perpetrators who are masculine-presenting. Thus, the labels of perpetrator and victim might influence perceptions of their gender presentation in a manner that emulates the ‘typical’ male-on-female case. Therefore, we expected that female perpetrators would be characterized by masculine traits, and male victims would be characterized by feminine traits (H^3.1^, H^3.2^).

Studies suggest that male victims are blamed more than female victims (Russell, 2018, Stanziani et al., 2018), presumably because they violate normative expectations of who can and who cannot be a victim of IPV. Thus, we expected that network models of prototypes involving a male victim would feature greater strength of victim blaming compared to models of prototypes involving a female victim (H^4^). Lastly, previous research has proposed physical differences between the perpetrator and the victim as a main predictor of the perceived severity of IPV (Hamby & Jackson, 2010). Therefore, we expected that the notion of physical superiority will be of greater strength in models with male perpetrators (H^5.1^) and of the greatest strength in the male-on-female prototype network (H^5.2^).

## Methods

### Participants

In total, 206 people entered the questionnaire on Prolific (prolific.com). Prolific is an online research platform that connects researchers with a large pool of pre-screened participants for survey and experimental studies. The platform helps with recruiting samples and managing participant payment logistics. It is suited for this type of research, as it allows us to reach the targeted demographic (i.e., U.S.- and U.K.-based adults) in an anonymous format, which is advantageous when asking about sensitive topics such as IPV. Of the 206 persons entering, 164 participants completed the questionnaire, of which 3 were excluded for giving irrelevant and/or uninterpretable answers. The final sample used for the analyses consisted of 161 participants, of which 149 responded to all 4 scenarios. The 12 participants who did not respond to all scenarios were retained, as their missing data were relatively evenly distributed across scenarios and did not substantially affect the sample size of any group. Participants had to be at least 18 years old and native English speakers to participate. The pre-screening for both age and language was provided by Prolific and by collecting from a U.S.- and U.K.-based sample pool. Our choice of an English-speaking sample was to mirror the demographics of previous studies, allowing for better comparison among them. Of the participants, 107 identified as female (66.5%), 53 as male (32.9%), and 1 as non-binary/other (0.6%). The participants’ ages ranged from 19 to 79 years (*M* = 39.92, *SD* = 11.93, Mdn = 38). Further, 138 participants indicated they were White (85.7%), 7 participants said they were Asian (4.4%), 6 were Black or African American (3.7%), and 10 participants indicated another race/ethnicity (6.2%). Finally, 139 participants identified as heterosexual or straight (86.4%), 10 indicated they were bisexual (6.2%), 10 said they were gay or lesbian (6.2%), and 2 indicated another sexuality (1.2%).

### Materials

The questionnaire was created using Qualtrics (qualtrics.com). All participants were asked to give open-ended responses to each of the four binary victim–perpetrator gender combinations, which were presented in randomized order. To prevent uncertainty about the type of responses expected, participants were given an example scenario including example answers, regarding typical assumptions about weddings. The example followed the same format as the actual items. Prior to answering the items, the World Health Organization’s (2021) definition of IPV was presented. The items asked participants to provide four open-ended answers for each scenario, referring to the victim, the perpetrator, the relationship, and the incident itself. Specifically, the participants were asked to provide stereotypes they think people ‘most commonly hold’ of the four binary gender combinations. Asking for commonly held beliefs in this manner was intended to reduce potential social desirability effects (viz. participants attempting to mitigate overt use of stereotypes to avoid being perceived as homophobic), while still prompting participants to provide a description of their mental representations of the IPV scenarios (see Dovidio & Gaertner, 2004, Moreno & Bodenhausen, 2001). After each IPV scenario, participants were asked to indicate on a five-point Likert scale how difficult it was to generate their responses (meta-cognition item). Like the global statistics of the network models, the meta-cognition item aimed to obtain a measure of prototype accessibility and richness. The entire questionnaire can be found here: osf.io/az24e.

### Procedure

Prior to the data collection, a pretest (*N* = 10) was conducted to test the questionnaire and get a rough idea of the quality and quantity of responses. No issues with the questionnaire were found. The pretest indicated that the responses would be sufficiently rich in content. Thus, the 10 responses from the pretest were included in the final sample. Following the pretest, the study was preregistered on the Open Science Framework (OSF) at osf.io/y3dr8.

Answering the questionnaire took 11 min on average and the participants were paid approximately £1.75 for their time. Due to fluctuating exchange rates, the budget of €500 for the data collection was sufficient to reach a sample size of around 160 participants. All participants were asked for informed consent before starting the questionnaire and demographic information was collected at the end.

Out of 164 participants, 12 participants provided responses for a different binary-gender combination than the one they were asked about in at least one of the scenarios. These cases were treated as responses to the gender combination the participant apparently responded to (rather than what they were actually presented). All such recodings were discussed and subsequently decided by two coders. The coded responses and further details on the recodings and exclusions can be found here: osf.io/7j4dv.

### Data Analysis

The data were analyzed using a quantitative-dominant mixed-methods design (Johnson et al., 2007), in which an initial qualitative phase generated the variables for subsequent quantitative modeling. All analysis scripts are available on GitHub: github.com/hannfei/ipv_network and archived on Zenodo (https://doi.org/10.5281/zenodo.20592952). First, participants responded to open-ended questions that allowed them to describe IPV in their own words. Their answers varied in length, ranging from a few bullet points to multiple sentences. The responses were processed using the *R* package *spacyr* (Benoit & Matsuo, 2020) and then subjected to a content analysis, in which the scenarios were systematically coded by human coders. To support the quality of this process, we followed the first three phases of Braun and Clarke’s (2006) approach to thematic analysis. Unlike a conventional thematic analysis, we did not cluster codes into broader themes. Instead, the individual codes served as the units of analysis for the quantitative phase. Next, these codes were transformed into binary indicators (present/absent) for each participant and prototype and used to estimate network models and conduct statistical tests of our hypotheses. In these network models, nodes represent the codes (i.e., features participants ascribed to the prototypes), and edges represent their co-occurrence, providing a visual and analytic summary of how different features are interrelated. Further details of the qualitative and quantitative procedures are provided in the sections below.

#### Qualitative Coding

To systematically code the content of the open-ended responses, we conducted a content analysis that was inspired by Braun and Clarke’s (2006) thematic analysis framework. That is, we followed only phases 1–3, as they were suitable for our analysis. Phases 4–6 are not applicable to our analysis, as they pertain to the creation of themes and the production of the final report. We performed a preliminary analysis of the pretest data (*N* = 10), to test the feasibility of the planned analysis approach and a codebook, to ensure consistent coding. The preliminary codes and categories (osf.io/hng5x) and the coding manual (osf.io/dj95v) can be found on the OSF. After familiarizing ourselves with the dataset (phase 1: reading and re-reading all responses and noting initial impressions; Braun & Clarke, 2006), the responses were pre-processed using the natural-language-processing tool *spacyr* (Benoit & Matsuo, 2020). That is, the responses were parsed by sentences, and words/sequences deemed irrelevant for the analysis were removed. We decided on a case-by-case basis which words to remove based on whether they contributed meaningful information to the analysis. For example, expressions such as ‘I think that (. . .)’ did not add analytical value. The parsed responses were then given to five human coders to generate the initial codes (phase 2; Braun & Clarke, 2006). The first author coded the entire dataset, and four additional coders each independently coded approximately 25% of the data. Coding was conducted independently in two rounds per coder, after which coders met in pairs to compare and discuss their codes, resolving any discrepancies by consensus. In the final step, to make the data more suitable for network analysis, the first and the second authors independently collapsed similar codes and identified broader categories. For example, codes describing different forms of physical abuse—such as punching or shoving—were collapsed into the same code, AE05 (aggravated (physical) abuse) and put under the category ‘Abuse’. We then met to discuss our codes and categories, until a consensus was reached. This procedure is analogous to phase 3 from Braun & Clarke (2006), with the exception that no themes—in the conventional sense—were created, as our aim was to retain detailed codes for the subsequent quantitative analyses, rather than develop higher-order themes.

#### Network Estimation

All quantitative analyses were conducted using *R* (Version 4.3.1; R Core Team, 2023) in RStudio (Posit Team, 2023). The codes derived from the qualitative analysis were transformed into binary variables: They were *either present* (1) or *not present* (0) in the participants’ responses for each of the scenarios. Polychoric correlation matrixes were calculated from the binary data and used as the input for the network estimation, as recommended by Epskamp and Fried (2018). Gaussian Graphical Models (GGM) were estimated for each of the four binary victim–perpetrator gender combinations using the *glasso* package (Friedman et al., 2019). The graphical least absolute shrinkage and selection operator (GLASSO; Friedman et al., 2008) performs regularization by estimating edge weights using penalized maximum (pseudo) likelihood, applying a penalty that shrinks weak or potentially spurious connections to zero. Gamma was set to .50 and the lambda ratio was set at .01. The estimated networks were visualized using the *qgraph* package (Epskamp et al., 2012).

Network comparison tests (NCTs) were performed to compare the global strength and density of the male-on-female prototype to the other prototypes. The permutation-based NCT creates a reference distribution to which the to-be-tested statistic is compared. If it exceeds what would be expected from a model of the same population (*p* < .05), the network is significantly different. Additional NCTs were performed to compare local statistics (i.e., centrality and strength) across the prototypes, to test specific hypotheses.

## Results

The responses of 161 participants were thematically coded and collapsed into 58 codes in 10 different categories pertaining to: The abuse, the circumstances, controlling behavior, dependency, gender, manipulation, victim blaming, and assumptions about the perpetrator, victim, and the relationship. Table 1 provides an overview of all the codes. A list detailing which codes were collapsed can be found on osf.io/d5uqx.

**Table 1.** List of Codes and Categories.

**AE**	**Abuse**	**PT**	**Perpetrator**
AE01	Physical abuse	PT01	Perpetrator is mean-spirited
AE02	Emotional abuse	PT02	Perpetrator lacks emotional control
AE03	Sexual abuse	PT03	Perpetrator is aggressive
AE04	Abuse not otherwise specified	PT04	Perpetrator is dominant
AE05	Aggravated (physical) abuse	PT05	Perpetrator is selfish
AE06	Abuse minimized	PT06	Perpetrator has low self-esteem
AE07	Less severe violence	PT07	Perpetrator is the ‘abusive type’
		PT08	Perpetrator is extroverted
**CS**	**Circumstances**	PT09	Perpetrator has psychological problems
CS01	Involvement of substances	PT10	Perpetrator is physically superior
CS02	Live under bad circumstances	PT11	Perpetrator is smart/powerful
CS03	Social isolation	PT12	Perpetrator is arrogant/confident
CS04	Have children		
		**RS**	**Relationship**
**CT**	**Controlling behavior**	RS01	Toxic relationship
CT01	Controlling behavior, not otherwise specified	RS02	Relationship is not equal
CT02	Jealousy and suspicion	RS03	Power imbalance in favor of the perpetrator
CT03	Limiting the victim’s social autonomy	RS04	Power imbalance in favor of the victim
CT04	Limiting the victim’s financial autonomy	RS05	Long-term relationship
		RS06	Uncommitted relationship
**DP**	**Dependency**	RS07	Non-romantic relationship
DP01	Dependency	RS08	Live together/See each other often
**GN**	**Gender**	**VT**	**Victim**
GN01	Traditional gender values	VT01	Victim is weak
GN02	Skepticism of women’s violence against men	VT02	Victim has limited social life
GN03	Victim is feminine/not masculine	VT03	Victim has low self-esteem
GN04	Perpetrator is masculine/not feminine	VT04	Victim is ashamed
		VT05	Victim is unassertive
**MP**	**Manipulation**	VT06	Victim is introverted
MP01	Manipulation not otherwise specified	VT07	Victim is kind-spirited
MP02	Perpetrator is manipulative	VT08	Victim has psychological problems
MP03	Hiding the abuse from others	VT09	Victim is scared, unable to self-defend
		VT10	Victim is social adept/assertive
**VB**	**Victim blaming**	VT11	Victim considers aspect of relationship positive
VB01	Victim blaming		
VB02	Blurring of victim/perpetrator		

### The Estimated Networks

**Figure 1.**
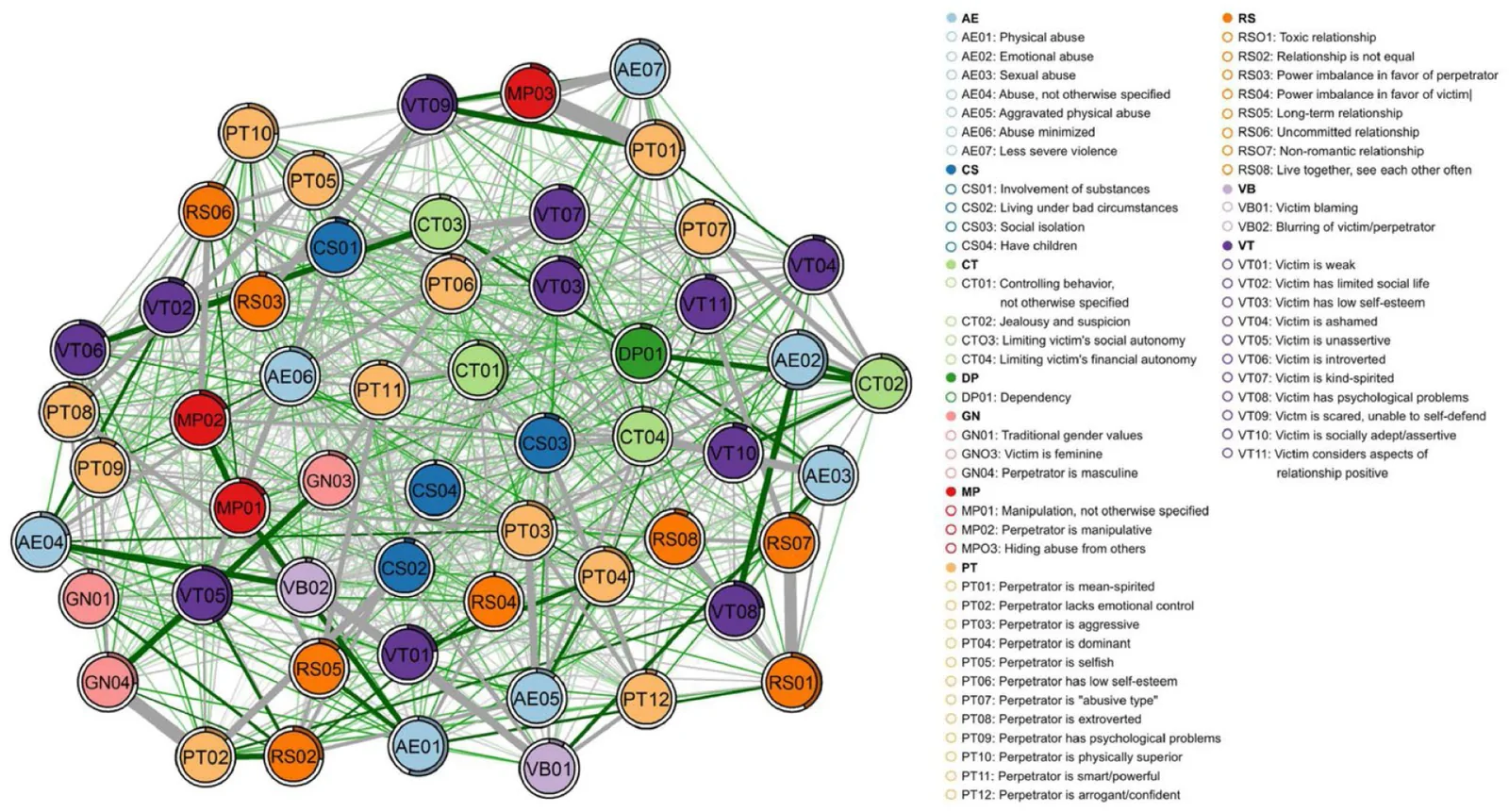
Estimated network of the female-on-female intimate partner violence prototype.

**Figure 2.**
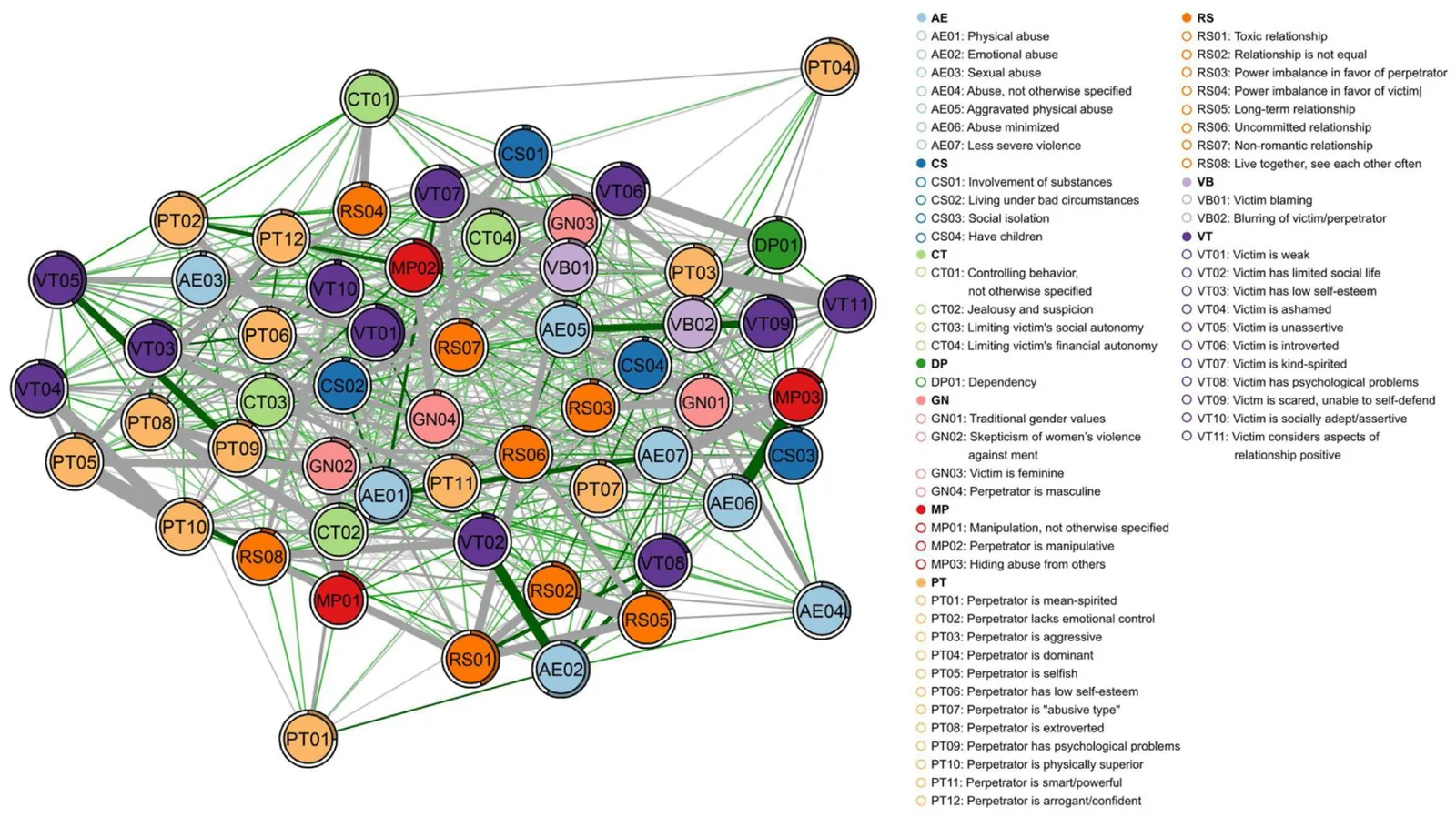
Estimated network of the female-on-male intimate partner violence prototype.

**Figure 3.**
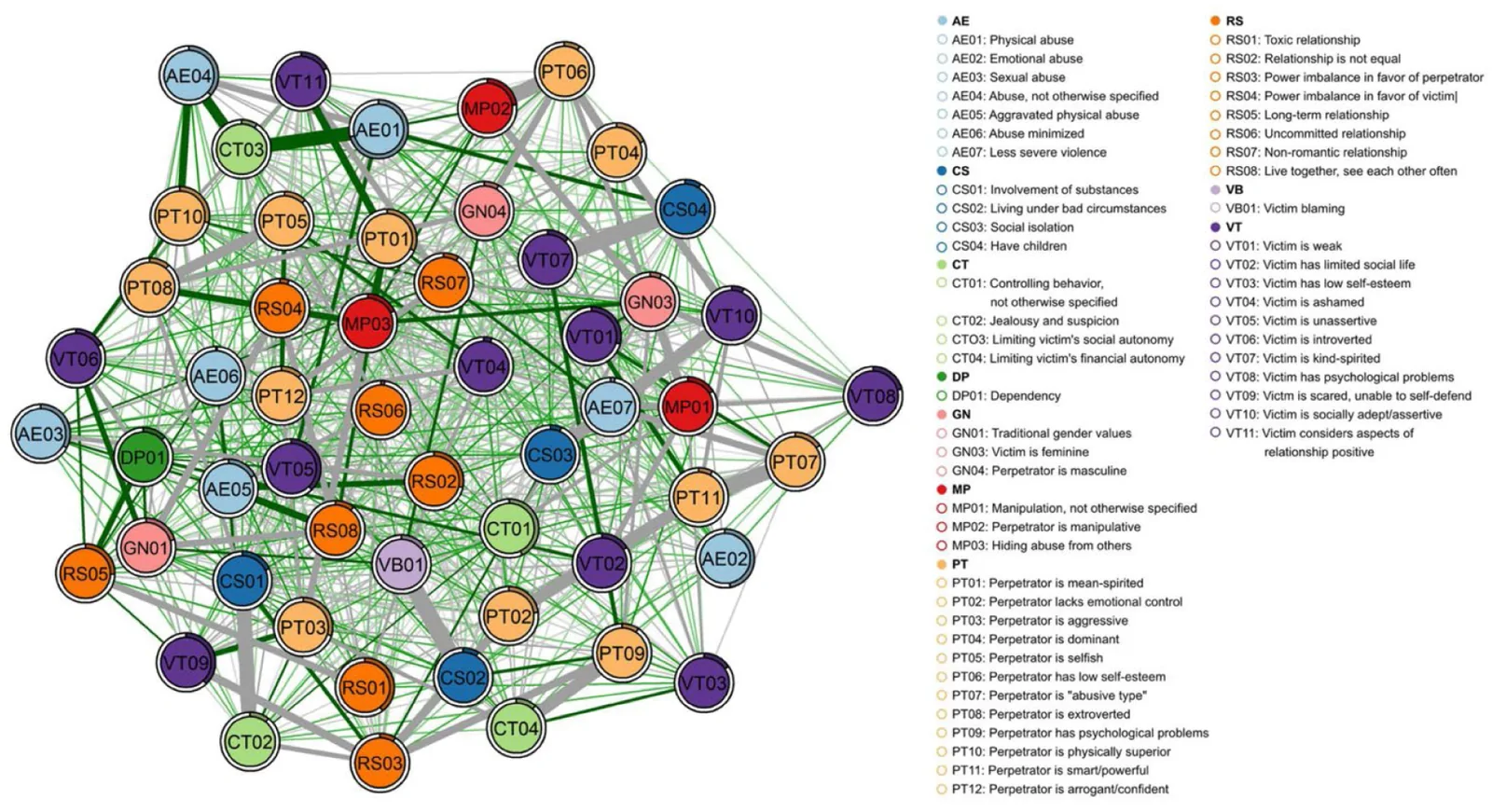
Estimated network of the male-on-female intimate partner violence prototype.

**Figure 4.**
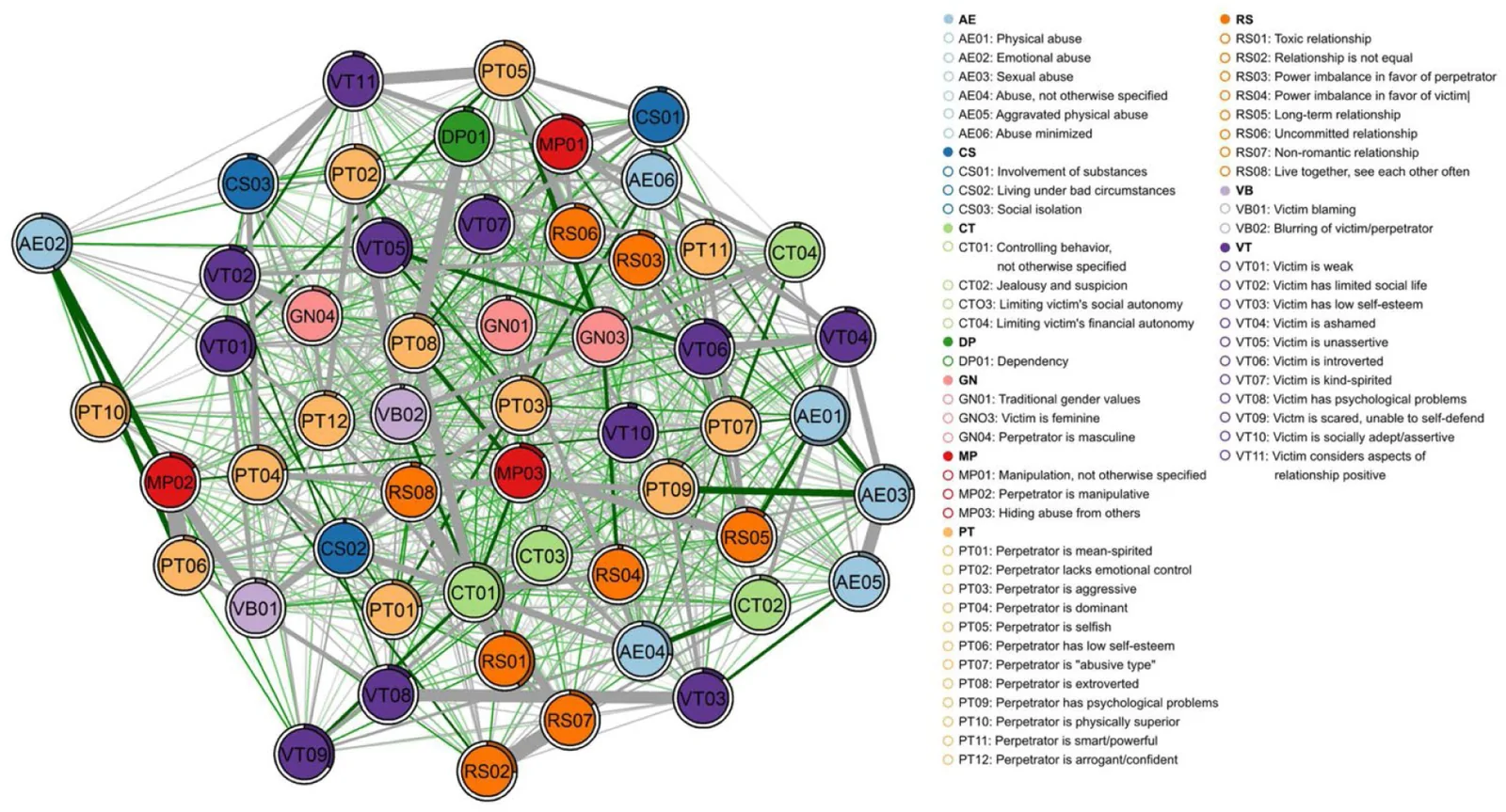
Estimated network of the male-on-male intimate partner violence prototype.

One network (GGM) was estimated for each scenario, representing the respective prototype. The centrality measures of the networks can be found at osf.io/a724q. The networks provide a statistical and visual overview of the content of the prototypes. The codes are represented by nodes, colored based on the categories they belong to (Figures 1–4; for full-resolution images, see github.com/hannfei/ipv_network/tree/main/figures). Partial correlations between the nodes are shown as edges between them. The thicker the edge, the stronger the correlation between the nodes. Gray edges indicate negative relationships (i.e., descriptions that contain one feature tend not to contain the other feature) and green edges positive relationships (i.e., descriptions that contain one feature tend to also contain the other feature). Because positive relationships indicate co-occurrence in descriptions, they can be substantively interpreted as indicating thematic consonance. In this context, negative relationships can be interpreted as indicating distinctiveness, since negatively related nodes tend not to co-occur in descriptions. Distinctiveness does not necessarily indicate that nodes have opposite meanings, however. Negatively related nodes could be thematically similar but mentioned in a mutually exclusive manner and alongside distinct sets of other features. Thus, negative relationships should be interpreted carefully and in context of other connected nodes.

As indicated by their density, the networks feature a conservative number of edges between their nodes (see Table 2). The colored parts in the circles around the nodes show the proportion of participants that included the respective code in their response to the scenario. The networks’ strength and density are similar across the prototypes (see Table 2). We performed network comparison tests (NCT) to assess the differences in global strength and global density between the male-on-female network and the other networks. We expected the male-on-female network to differ significantly in global strength and density (H^1.1^, H^1.2^), as this scenario should be the most familiar to participants, prompting a greater variety of responses. However, as can be seen in Table 3, no significant differences in strength and density were found. The prototypes are similarly coherent.

**Table 2.** Global Statistics of the IPV Networks.

	FF	FM	MF	MM
Strength	4.724	4.077	4.491	4.963
Density	.539	.438	.491	.557

*Note.* FF: female-on-female; FM: female-on-male; MF: male-on-female; MM: male-on-male.

**Table 3.** *p*-values of the NCTs for Global Strength and Density.

	MF vs. FM	MF vs. FF	MF vs. MM
Global strength	.359	.678	.708
Density	.434	.732	.661

*Notes.* MF: male-on-female; FM: female-on-male; FF: female-on-female; MM: male-on-male.

### The Proportional Presence of Codes

**Figure 5.**
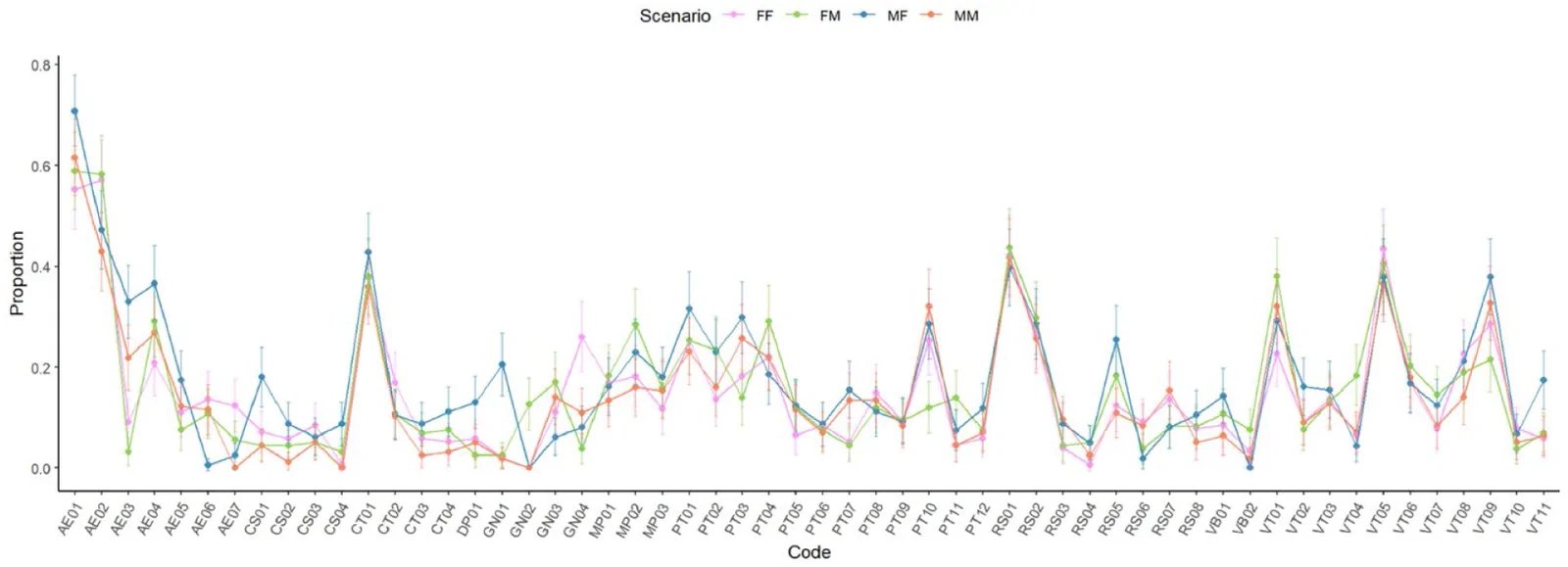
Proportional presence of the codes in the answers for each scenario, with 95% CIs.

The frequency distribution of the codes across the networks shows no large differences in the proportional presence of the codes (Figure 5). All networks follow a highly similar pattern, with only a few differences (for details, see osf.io/xhau6). Notably, there is a stark difference between the perpetrator in the female-on-female scenario and the perpetrator in the female-on-male scenario. While both are women, the perpetrator in the lesbian scenario is described as masculine (GN04) more often. In contrast, the perpetrator being manipulative (MP02), dominant (PT04), and smart/powerful (PT11) is more frequently attributed to the female perpetrator in the female-on-male scenario. This contrasts with the low assignment of physical superiority to the female perpetrator (PT10) in this scenario, compared to the perpetrators in the other scenarios.

### Comparisons of Scenarios

Although the NCTs indicated similar levels of coherence across the scenarios, participants’ meta-cognitive appraisals paint a different picture. As expected in hypothesis 1.3, paired-samples *t*-tests indicate that the male-on-female scenario was significantly easier to answer (*M* = 1.91, *SD* = 0.88) than the other scenarios (see Table 4).

**Table 4.** Paired *t*-Test Results for Ratings of Response-Difficulty Across Scenarios.

Comparison	Mean_non-MF_ (*SD*)	*t*-value	*df*	*p*-Value
MF–FF	2.98 (1.19)	−11.30	160	*p* < .001
MF–FM	2.75 (1.15)	−9.25	160	*p* < .001
MF–MM	2.93 (1.11)	−11.55	158	*p* < .001

*Note.* MF: male-on-female; FF: female-on-female; FM: female-on-male; MM: male-on-male.

**Figure 6.**
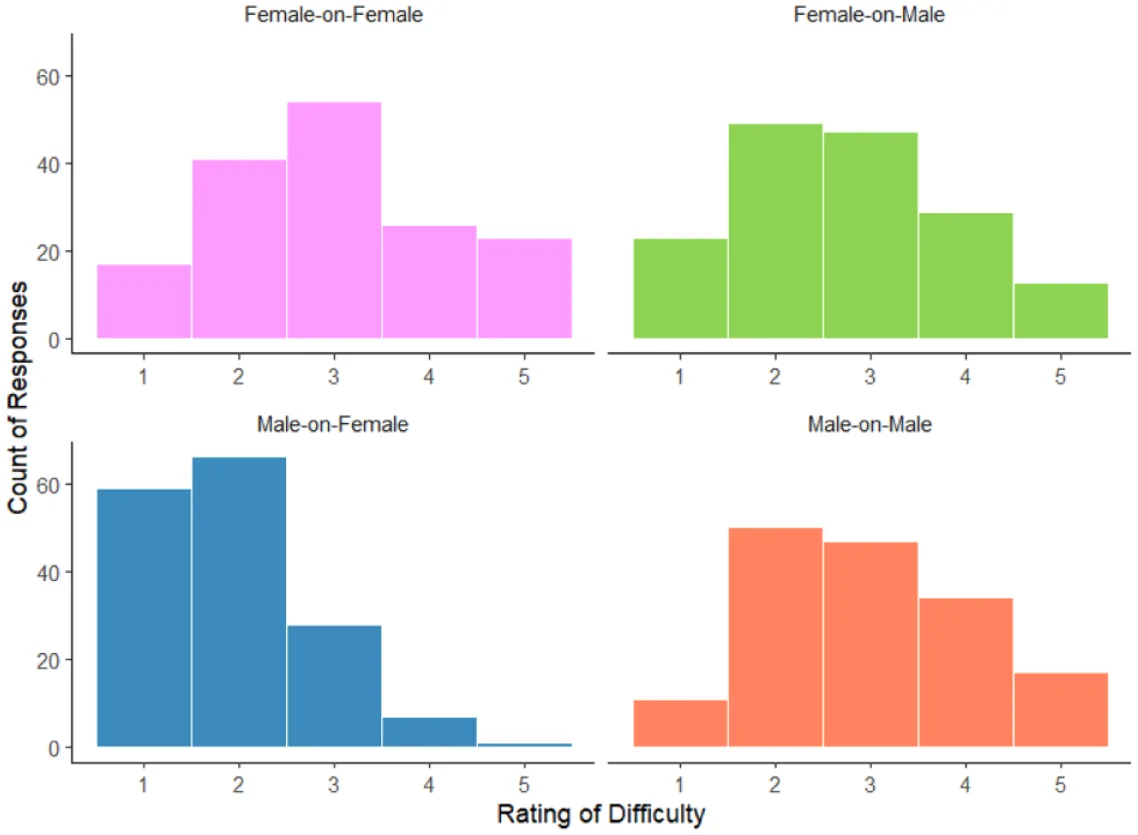
Frequency distributions of response-difficulty ratings across scenarios.

To further explore this finding, we also compared the female-on-female, male-on-male, and female-on-male scenarios to each other. The difficulty rating between the female-on-female and the female-on-male scenario differs significantly, *t*(160) = 2.51, *p* = .013, such that the participants thought it was easier to generate responses for the female-on-male scenario. Similarly, the difficulty rating between the male-on-male and the female-on-male scenario differs significantly, *t*(158) = 2.06, *p* = .041, once again indicating that participants thought it was easier to generate responses for the female-on-male scenario. There was no significant difference between the two same-sex scenarios, *p* = .722. A comprehensive table of the results can be found at osf.io/uxmpw. Figure 6 shows the frequency distribution of the ratings of difficulty across scenarios (1 = *not at all difficult*; 5 = *very difficult*).

As the literature shows that the male-on-female scenario is considered to be the most severe, we expected greater local strength of the physical abuse nodes in the male-on-female network compared to the other networks (H^2^). In other words, we assumed physical abuse would be more strongly connected to the other features in the male-on-female prototype than in the other prototypes. Thus, NCTs were performed to compare the local strength of the physical abuse nodes (AE01, AE05) of the male-on-female network to the other networks. Contrary to our assumption, no significant differences were found: MF versus FM, *p* = .416; MF versus FF, *p* = .647; MF versus MM, *p* = .102.

To assess the assumption that female perpetrators and male victims are assigned opposite gendered characterizations (H^3.1^; H^3.2^), exact cell-wise analyses of two-way contingency tables were performed, based on Fisher’s fixed margins model (Bergman & El-Khouri, 1987). This analysis tests whether a specific frequency is different from what could be expected in an independence model. The independence model assumes that every arrangement of the gendered traits within the data is equally probable.

Aligning with Hypothesis 3.1, female perpetrators with female victims were assigned significantly more masculine traits than would be expected in an independence model (*OR* = 2.15, *p* < .001). In contrast to the hypothesis, female perpetrators with male victims were assigned fewer masculine traits (*OR* = 0.31, *p* < .001). Neither male perpetrator of the other scenarios was assigned significantly more or fewer masculine traits.

Male victims of female perpetrators were assigned significantly more feminine traits, as predicted by Hypothesis 3.2 (*OR* = 1.41, *p* = .048). Opposing the hypothesis, male victims of male perpetrators were not assigned more feminine traits (*p* = .224). Furthermore, significantly fewer feminine traits were assigned to the female victim of the male perpetrator (*OR* = 0.51, *p* = .013). The male-on-female scenario was also assigned the fewest gendered traits overall. A comprehensive table of these findings can be found on osf.io/nq6c3.

We assumed that victim blaming would be of greater local strength in scenarios with male victims (H^4^). That is, we expected victim blaming to be stronger connected to the other features in prototypes with male victims, indicating a greater influence of this feature on the overall structure of the prototype. To test this, network comparison tests (NCTs) were performed to compare the local strength of victim blaming (VB01) in scenarios with a male victim (FM, MM) to scenarios with a female victim (MF, FF). The only significant difference in the strength of victim blaming was found between the male-on-male and the male-on-female scenario (*p* < .001), providing partial support for our hypothesis. This suggests a greater centrality of victim blaming in the male-on-male network than in the male-on-female network. The other comparisons did not indicate significant differences: FM versus MF, *p* = .909; FM versus FF, *p* = .297; MM versus FF, *p* = .727.

Additional NCTs were performed comparing the local strength of the perpetrator’s physical superiority (PT10) in scenarios with a male perpetrator (MM, MF) to scenarios with a female perpetrator (FF, FM). That is, how strongly this feature was connected to the other features in each network. We assumed this connection would be stronger in scenarios with a male perpetrator (H^5.1^) and the strongest in the male-on-female scenario (H^5.2^). However, no significant differences in local strength between scenarios with male and female perpetrators were found: MF versus FF, *p* = .374. MF versus FM, *p* = .384. MM versus FF, *p* = .838. MM versus FM, *p* = .170. When comparing the local strength of physical superiority of the male-on-female network to the others, a significant difference in local strength was observed between the male-on-female and the female-on-male scenario, *p* = .021, providing partial support for Hypothesis 5.2. No other comparison indicated significant differences: MF versus FF, *p* = .342. MF versus MM, *p* = .118.

### Exploratory Analyses

The similarities in global statistics between the networks were unexpected. However, a visual inspection of the networks indicates that the models are not identical (see Figures 1–4). They might be similarly coherent, but they are not structurally equivalent. To assess this assumption, multiple-group comparisons between the networks were performed. The *psychonetrics* package in *R* was used to refit the models using maximum likelihood estimation and to calculate fit statistics (Epskamp, 2023). The adjacency matrix of each network was extracted and fitted to all datasets, in a process comparable to a test of factorial invariance. A comparison of the Bayesian information criterion (BIC) indicates that the models clearly differ: The BIC for each scenario’s model increased substantially whenever the model was fit to the data for another scenario (see Table 5).^1^

**Table 5.** Model-Fit Statistics of the Multiple-Group Comparisons Between the Networks.

Data	BIC	Data	BIC
*Female-on-female model fitted to*		*Male-on-female model fitted to*	
FF	13,825.76	MF	17,303.83
FM	23,218.74	FF	23,165.26
MF	22,705.88	FM	23,917.22
MM	22,110.17	MM	23,236.13
*Female-on-male model fitted to*		*Male-on-male model fitted to*	
FM	17,701.94	MM	14,978.40
FF	24,076.80	FF	22,268.64
MF	23,795.85	FM	22,944.24
MM	23,773.99	MF	22,351.37

*Note.* MF: male-on-female; FF: female-on-female; FM: female-on-male; MM: male-on-male.

## Discussion

The present study assessed the structure of IPV prototypes using a data-driven approach, allowing for more spontaneous participant responses and imposing fewer predetermined constraints on the descriptions of IPV compared with previous research. One striking finding is that the prototypes for the different scenarios do not seem to differ substantially in their basic content (i.e., the rates at which different codes occurred), but that the configuration of that content (i.e., the correlations between codes) differs vastly between the gender combinations. To the extent that the meaning of a code is determined by the content with which it is correlated, this finding suggests that the same feature of IPV (e.g., physical violence) can take on dramatically different meanings depending on the genders of the victim and perpetrator. The statistical analyses of the global and local characteristics of the network models of each gender combination support this overarching interpretation.

### A Lack of Global Differences Between Prototypes

Differences in global strength and density—that is, differences in how many links there are between features and how strongly those features are connected—would indicate a difference in the coherence of the prototypes and how the features within each prototype interact. As people seem to use the ‘typical scenario’ (male-on-female IPV) as a reference point and are more familiar with its prototype (Dagenbrink et al., 2023; Whitehead et al., 2021), we expected this scenario to show a more tightly connected pattern of features. Surprisingly, no significant difference between the prototypes in terms of global strength and density was observed. A look at the frequency distributions shows a very similar pattern, with only a few exceptions. However, while the prototypes are similarly coherent, they are not identical, visually or statistically, as indicated by the model fit tests. Moreover, the participants found it significantly harder to generate responses for the ‘atypical’ scenarios than for the ‘typical’ male-on-female scenario.

### Fine-Grained Differences Between Prototypes

The confirmatory analysis aimed to assess differences between the networks in the extent to which particular features were connected to the rest of the network (local strength). This made it possible to identify which features were more central in one prototype than in the others and therefore had a greater influence on the overall structure of the prototype. We find that victim blaming was more central only in the male-on-male scenario compared to the male-on-female scenario. While this supports the notion in the literature that men violate the perception of who can and cannot be a victim (Hine et al., 2022; E. P. Seelau et al., 2003), the same does not seem to be true for the male victim in the female-on-male scenario. Here, no significant difference in the strength of victim blaming was observed, compared to the scenarios with female victims. It seems like the notion that ‘men should be able to fight back’ is true for men who become the victims of other men, but not for men who become victims of women. A potential, yet speculative, explanation is that men ‘fighting back’ against women is stigmatized more and might blur the lines between victim and perpetrator.

In the literature, the severity of the abuse is consistently rated highest for the male-on-female scenario, even when the incident described is the same across all binary-gender combinations (Russell, 2018; Stanziani et al., 2018). One explanation that has been presented for these differences in perceived severity is the perceived physical superiority of the perpetrator (Hamby & Jackson, 2010). If the assumption is that a stronger perpetrator can do more harm, and men tend to be physically stronger than women, this might explain the hierarchy often found in ratings of severity. In the present study, the only significant difference in how strongly physical superiority is connected to the other features in the prototype was found between the male-on-female scenario and the female-on-male scenario. This difference in local strength does suggest that physiological differences play a role in the perceptions of gender differences in IPV between the male-on-female and female-on-male scenario. It does not, however, seem to play a role in the different perceptions between the male-on-female and the same-sex scenarios. Furthermore, an explanation based on physiological differences cannot account for the fact that male-on-female (vs. female-on-male) IPV is perceived as more severe across both physical and psychological abuse (Ahmed et al., 2013). A potential explanation in support of the importance of physical differences is the assumption that psychological abuse has the potential to develop into physical violence. However, participants rarely included this idea in their descriptions of IPV, making the assumption unlikely and unfit for empirical testing.

One might argue that physiological differences only matter in scenarios that are presumed to involve more physical abuse than others. This would require differences between the networks in how strongly physical abuse is connected to the other features. In particular, physical abuse would need to be more strongly connected to the other features in the male-on-female prototype, as the literature suggests that this scenario is perceived as the most severe. However, no significant differences in the importance of physical abuse between the networks were found. Furthermore, there is no direct link between physical abuse and the physical superiority of the perpetrator in the male-on-female network, hinting at no direct connection between them.

Despite the lack of global differences between the networks in how many links there are between features and how strongly those features are connected, participants’ assessments of how difficult it was to generate responses indicate that the male-on-female scenario is perceived to be the most ‘typical’: The male-on-female scenario was considered to be the easiest to answer, followed by the female-on-male scenario, which in turn was rated significantly easier to answer than the same-sex scenarios. There was no significant difference between the same-sex scenarios. This suggests that assumptions about heterosexual IPV seem to be generally more easily accessible than assumptions about same-sex IPV.

The assignment of gendered traits to some of the victims and perpetrators further supports the notion that the male-on-female scenario offers a baseline for the construction of the other prototypes. That is, some ‘atypical’ cases seem to be gendered in a manner that emulates the ‘typical’ male-on-female scenario. A woman being a victim is consistent with the gender roles in the typical scenario, thus, no ‘correction’ of the gendered traits of the victim in the female-on-female scenario is needed. In contrast, the male victim in the female-on-male scenario is assigned more feminine traits as an assimilation with the typically female victim role. In a similar fashion, the female perpetrator in the female-on-female scenario is assigned more masculine traits to assimilate perceptions with the typical male-perpetrator role.

### Limitations and Future Directions

Network models need large datasets to estimate stable networks, especially when a high number of nodes is present (Blanken et al., 2022). Keeping the sample size low was a conscious decision made under the limitations of the time and resources available. However, the reliability of the estimated networks in this study and their generalizability can be improved with a larger sample. It is also worth noting that the sample was mainly composed of heterosexual participants, and a sample with diversity in terms of sexual orientation might warrant different results. Similarly, most participants in our sample identified as White, despite the questionnaire being open to all ethnicities. We also recruited a U.S.- and U.K.-based sample to align with the context of most previous studies. The sample may also be subject to some self-selection bias, as Prolific users are individuals who choose to sign up for online research, either due to an interest in research or to receive small financial compensation. In addition, the example answers presented at the start of the questionnaire referred to a male-on-female wedding, which may have prompted participants to use similarly gendered framings in their responses. Future work would benefit from more ethnically diverse, intersectional samples and could examine whether more neutral examples lead to differences in perceptions of IPV.

A network model can become quite complex, and interpreting the implications of the connections can be challenging. As the differences between the prototypes seem to be within their configuration, and not their content, future research needs to be done to gain more insights into the internal structure of the networks. Instead of looking at the overall strength of certain nodes across the networks, future research can focus more on the edges between certain nodes of interest and how they might differ between the networks. This might offer insights into the more complex dynamics within the prototypes.

### Conclusion

The main aim of the present study was to characterize people’s beliefs and attitudes about gendered prototypes of IPV using network models. The links between features (i.e., specific characteristics of IPV) are interpretable as associated expectations, such that the presence of a characteristic implies the potential presence of other connected characteristics, and these connections vary between gender combinations. To the extent that these models adequately represent people’s expectations of IPV with different gender combinations of victims and perpetrators, they can be used to derive predictions of people’s perceptions and judgments. For example, visual inspection of the network models suggests that the presence of physical abuse would tend to make people more frequently expect that the perpetrator also limits the victim’s social autonomy in male-on-female IPV compared to female-on-male IPV. Future research might fruitfully examine such model-derived predictions and also make efforts to model prototypes in different populations. Such an approach would ensure that research on IPV perceptions is based on the content and structure of people’s actual beliefs, rather than on predetermined assumptions.

## Notes

1. The GLASSO estimation procedure, which works to minimize the BIC, set many edges to 0. The modification indices implied that model fit would substantially improve with more edges added, suggesting that the BIC forced a conservative solution. Consequently, the analytical approach may be too conservative for many of the model fit statistics to be interpreted. However, the BIC remains a reasonable fit statistic to compare these network models, since this was the value the estimation procedure attempted to optimize.
